# Endophytic *Streptomyces laurentii* Mediated Green Synthesis of Ag-NPs with Antibacterial and Anticancer Properties for Developing Functional Textile Fabric Properties

**DOI:** 10.3390/antibiotics9100641

**Published:** 2020-09-24

**Authors:** Ahmed M. Eid, Amr Fouda, Gniewko Niedbała, Saad El-Din Hassan, Salem S. Salem, Abdullah M. Abdo, Helal F. Hetta, Tharwat I. Shaheen

**Affiliations:** 1 Department of Botany and Microbiology, Faculty of Science, Al-Azhar University, Nasr City, Cairo 11884, Egypt; aeidmicrobiology@azhar.edu.eg (A.M.E.); Saad.el-din.hassan@umontreal.ca (S.E.-D.H.); salemsalahsalem@azhar.edu.eg (S.S.S.); Abdullah.abdo@azhar.edu.eg (A.M.A.); 2 Department of Biosystems Engineering, Faculty of Environmental Engineering and Mechanical Engineering, Poznań University of Life Sciences, Wojska Polskiego 50, 60-627 Poznań, Poland; gniewko@up.poznan.pl; 3 Department of Medical Microbiology and Immunology, Faculty of Medicine, Assiut University, Assiut 71515, Egypt; helalhetta@aun.edu.eg or; 4 Department of Internal Medicine, University of Cincinnati College of Medicine, Cincinnati, OH 45267-0595, USA; 5 National Research Centre, El-Behouth St., Dokki, P.O. Giza 12622, Egypt; ti.shahin@nrc.sci.eg

**Keywords:** silver nanoparticles, endophytic actinomycetes, green synthesis, cotton fabrics, antibacterial

## Abstract

Improvement of the medical textile industry has received more attention recently, especially with widespread of microbial and viral infections. Medical textiles with new properties, such as bacterial pathogens self-cleaning, have been explored with nanotechnology. In this study, an endophytic actinomycetes strain of *Streptomyces laurentii* R-1 was isolated from the roots of the medicinal plant *Achillea fragrantissima.* This is used as a catalyst for the mediated biosynthesis of silver nanoparticles (Ag-NPs) for applications in the textile industry. The biosynthesized Ag-NPs were characterized using UV-vis spectroscopy, Fourier transform infrared (FT-IR), transmission electron microscopy (TEM), and X-ray Diffraction (XRD), which confirmed the successful formation of crystalline, spherical metal nanoparticles. The biosynthesized Ag-NPs exhibited broad-spectrum antibacterial activity. Our data elucidated that the biosynthesized Ag-NPs had a highly cytotoxic effect against the cancerous caco-2 cell line. The selected safe dose of Ag-NPs for loading on cotton fabrics was 100 ppm, regarding their antibacterial activity and safe cytotoxic efficacy. Interestingly, scanning electron microscope connected with energy dispersive X-ray spectroscopy (SEM-EDX) of loaded cotton fabrics demonstrated the smooth distribution of Ag-NPs on treated fabrics. The obtained results highlighted the broad-spectrum activity of nano-finished fabrics against pathogenic bacteria, even after 5 and 10 washing cycles. This study contributes a suitable guide for the performance of green synthesized NPs for utilization in different biotechnological sectors.

## 1. Introduction

Nanotechnology has rapidly increased worldwide and been integrated into various biotechnological applications, such as the medicine, food, smart textiles, environmental hazard remediation, and agricultural sectors [1,2,3,4]. The prevalent usage of nanoparticles (NPs) may be attributed to new behaviors that arise at the nanoscale encompassing distinctive optical, chemical, electrical, and catalytic properties; excellent chemical stability; a wild variety of biological activities; and biocompatibility [5,6,7]. Metals and metal oxide nanoparticles have been fabricated by different approaches, including chemical, physical, and biological methods. However, biological methods are preferred over chemical and physical ones due to their cost-effectiveness, eco-friendliness, non-harsh conditions, and in not producing hazardous by-product compounds [8,9]. The biological synthesis of NPs can be accomplished by using different biological catalysts, such as bacteria, fungi, yeast, actinomycetes, plants, and algae [10].

The spread of microbial infections in health care, such as hospitals, is a severe problem that threatens human health. Textiles are considered one of the main factors responsible for the spread of infections between patients and others. Therefore, it is necessary to develop medical textiles that have the ability to reduce the spread of microbial infection by introducing resistance to the medical textiles of pathogenic bacteria. Nanoparticles provide effective tools to accomplish this hypothesis through incorporation into textiles during manufactures [3,11,12].

Endophytic actinomycetes are organisms that colonize internal plant tissues without any symptoms of diseases [13,14]. Endophytic actinobacteria, especially *Streptomyces* spp., have the ability to secrete different secondary metabolites, which are used as a biocatalysts to fabricate metal and metal oxide NPs such as Cu-NPs, CuO-NPs, Ag-NPs, and Au-NPs [15,16,17,18].

In the view of the medical field, silver NPs (Ag-NPs) have wide applications, such as skin ointments to prevent the infection of burns and wounds [19], to reduce bacterial infections [20,21]; biomedical diagnostic tools [22], and in the development of medical textiles [4]. Ag-NPs have been fabricated via chemical and physical methods; however, biological synthesis using bacteria [23], fungi [24,25], and actinomycetes [17] is preferred. 

Recently, the incorporation of Ag at the nanoscale to the textile industry has acquired increasing awareness due to new characteristics discovered for nanomaterials. Therefore, this study explored the efficacy of endophytic *Streptomyces laurentii* isolated from medicinal plant *Achillea fragrantissima* as a biocatalyst for the green synthesis of Ag-NPs. The characterization of the green synthesized Ag-NPs was assessed, and the biological activity of Ag-NPs, including the antibacterial activity and cytotoxicity, were investigated. Finally, the safe dose of green synthesized Ag-NPs that can be loaded onto cotton fabrics was determined and we explored the new features of the fabrics due to the Ag-NPs coating.

## 2. Results and Discussion 

### 2.1. Isolation and Identification of Endophytic Actinobacteria

**Figure 1:**
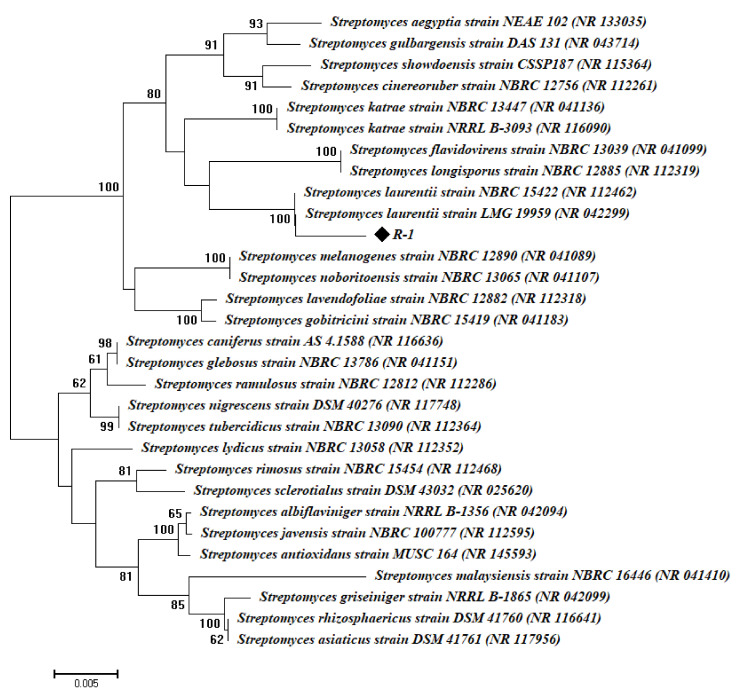
Phylogenetic tree of endophytic actinomycetes strain based on the 16S rRNA sequences analysis. Symbol ◆ indicates 16S rRNA fragments obtained from the current study. The analysis was completed with MEGA 6 using the neighbor-joining method.

Endophytic microorganisms, particularly actinomycetes, are known for their extracellular biologically active compounds, particularly those isolated from medicinal plants [26]. In this regard, root samples of the medicinal plant *A. fragrantissima*, located in Saint Katherine, South Sinai, Egypt, were collected. Only one actinomycete (R-1) was isolated from the root samples, and the molecular identification based on 16S rRNA gene sequencing showed that the R-1 isolate was closely related to *Streptomyces* sp. with a similar percentage of 99% to *Streptomyces laurentii* (accession number: NR042299) and identified as *Streptomyces laurentii* (R-1) (Figure 1). To date, this is the first report for the isolation of *Streptomyces laurentii* as endophytes from the medicinal plant of *A. fragrantissima* and used as a biocatalyst for the green synthesis of Ag-NPs. 

### 2.2. Biosynthesis of Silver Nanoparticles 

The green synthesis of nanoparticles using actinomycetes, fungi, bacteria, algae, and plants has better advantages than those of chemical and physical methods as they are environmentally safe and energy-saving; therefore, these biogenic nanoparticles can be used in various biotechnological and biomedical applications [27]. Actinomycetes, especially *Streptomyces*, are considered one of the best nominations for the biological production of nanoparticles on a large scale with low costs due to their high biomass production and rapid growth, in addition to being environmentally friendly [28]. Endophytic actinobacteria produce extracellular active metabolites, such as proteins and enzymes, that are involved in the biogenic fabrication of nanoparticles [16].

**Figure 2:**
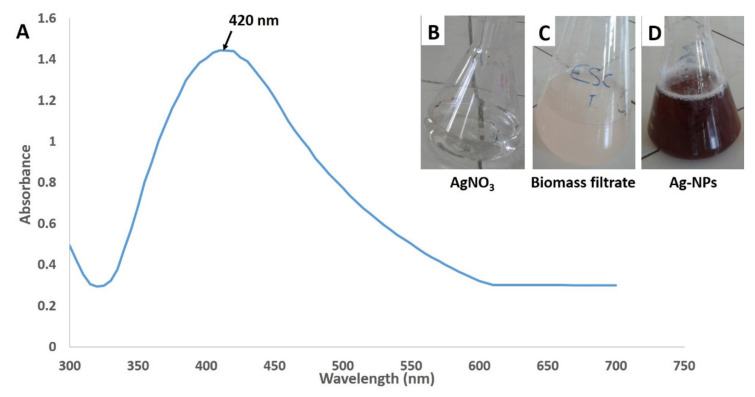
Ultraviolet-visible (UV-vis) spectroscopic analysis and the color change of silver nanoparticles (Ag-NPs) synthesized by endophytic *S. laurentii* R-1. (**A**) the UV-vis spectroscopy; (**B**) the AgNO_3_ solution; (**C**) the *S. laurentii* biomass filtrate, and (**D**) the biosynthesized Ag-NPs.

In the current study, the actinomycetal mediated synthesis of nanoparticles was optimized by adding 1 mM pure AgNO_3_ solution to the washed cell-free extract, incubated for 24 h, at 35 °C (pH = 9). The extracellular synthesis of nanomaterials was inferred by the color of the liquid shifting from colorless to yellowish-brown (Figure 2B–D). Silver nitrate solution and/or biomass filtrate were tested as a negative control and did not exhibit any color change after the incubation periods. The reduction of Ag^+^ to Ag^0^ could be attributed to an electron released as a result of reducing NO_3_ to NO_2_ by metabolites involved in the biomass filtrate [29,30]; therefore, Ag-NPs color intensity was correlated with the number of reduced silver ions.

The spectroscopic analysis confirmed the biogenic synthesis of Ag-NPs, which displayed a surface plasmon resonance (SPR) with a specific sharp peak at 420 nm, which was characteristic for the spherical Ag-NPs (Figure 2A) [31]. Fouda et al. [17] reported that the SPR range for Ag-NPs synthesized by endophytic *Streptomyces* spp. was 440–450 nm and that the standard SPR peak for Ag-NPs synthesized by the green approach was from 400 to 450 nm [32].

### 2.3. Characterization of Biosynthesized Silver Nanoparticles

#### 2.3.1. Fourier Transform Infrared (FT-IR) Analysis

**Figure 3:**
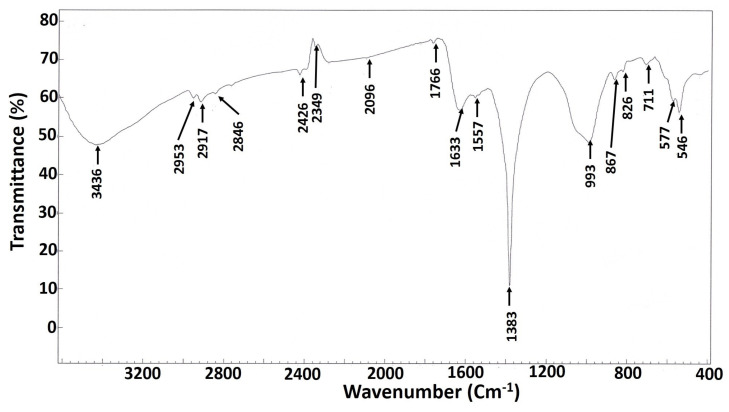
Fourier transform infrared (FT-IR) spectrum of the Ag-NPs derived from endophytic *S. laurentii* R-1.

FT-IR analysis was performed to identify the functional components involved in the capping, reduction, and stability of biologically fabricated Ag-NPs, which may be associated with several chemical bonds in the *Streptomyces* culture filtrates. The FT-IR spectral analysis for the synthesized Ag-NPs showed a broad peak at 3436 cm^−1^ (Figure 3), which is characteristic to N–H aliphatic primary amine [33], the peak at 2953 cm^−1^ is assigned to the carboxylic acid (OH) stretch [34], while the peak at 2917 cm^−1^ indicates the -CH_2_ group [35]. The band at 2349 cm^−1^ is compatible with the C-H asymmetric stretching vibration for aliphatic groups [36]. The spectrum peak at 2096 cm^−1^ corresponds to the (OH) stretch of carboxylic acid. Bands at 1633 and 1557 cm^−1^ are correlated with the binding of the amide I band of protein with a (N–H) stretch [34]. The observed band at 1383 cm^−1^ can be set to C–N stretching vibrations for aromatic and aliphatic amines [37,38]. The peak recorded at 867 cm^−1^ indicates the amide IV (OCN) stretch bending for protein. Meanwhile, the (S–S) stretch band of protein was observed at 577 cm^−1^ can be referred to a carbohydrate moiety [39]. The peak observed at 826 cm^−1^ is assigned for alkenes C–H vibration [40]. The presence of alkene (=C–H bending) was inferred by the peak recorded at 546 cm^−1^ (Figure 3). The current results are consistent with previous reports, which concluded that proteins play a fundamental role in the formation of silver nanoparticles and act as a stabilizer and capping agent for biologically produced nanoparticles [41,42]. The carbonyl group of amino acids and proteins was shown to increases the stability of nanoparticles when bound to them [35]. 

#### 2.3.2. Transmission Electron Microscopy (TEM) Analysis

**Figure 4:**
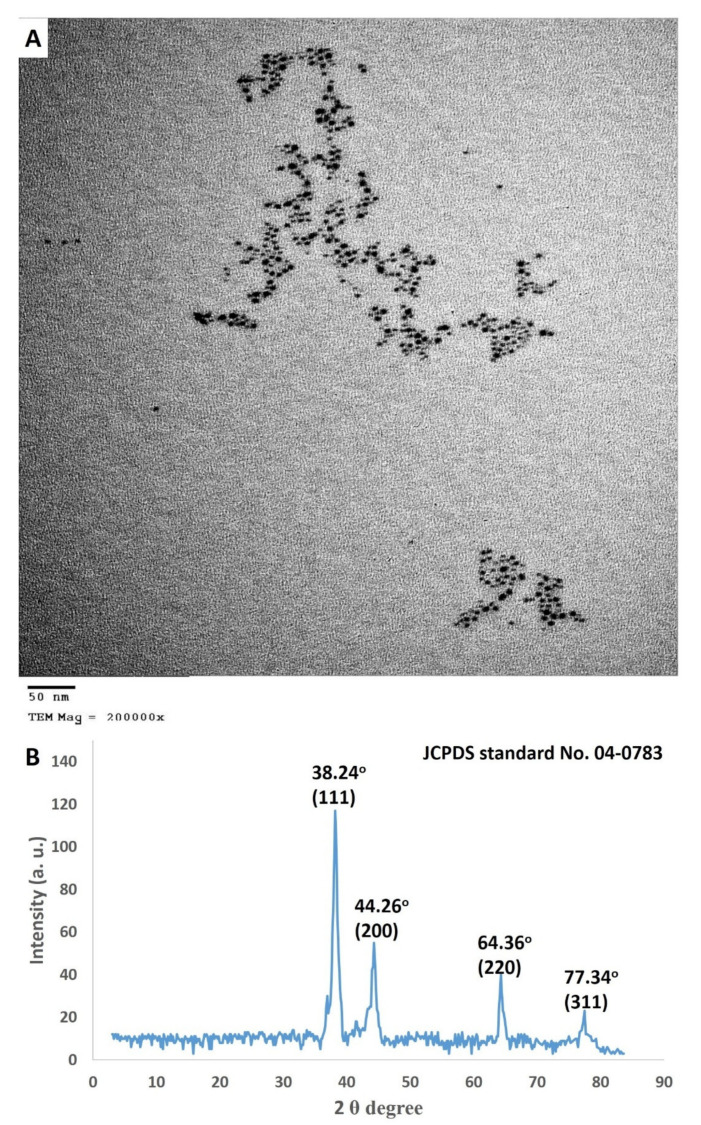
(**A**) Transmission eelectron microscopy (TEM) image showing the spherical shape with sizes ranging from 7 to 15 nm, and (**B**) X-ray diffraction (XRD) spectra exhibiting the crystalline nature of Ag-NPs synthesized by endophytic *S. laurentii* R-1. at specific 2 θ values.

The biotechnological and biomedical applications of NPs depend on their size and shape; a smaller size is better than a bigger size in applications [4]. In the current study, the TEM micrographs confirmed the formulation of anisotropic monodispersed bio-fabricated Ag-NPs, which were spherical and of varying sizes ranging from 7 to 15 nm (Figure 4A). In addition, the biologically prepared nanoparticles were well dispersed without any agglomeration or disparity in their morphology. In line with our results, Hamouda et al. [43], who reported the bio-fabrication of stable, monodispersed Ag-NPs from the aqueous extract of cyanobacteria.

#### 2.3.3. X-ray Diffraction (XRD) Analysis

The structural properties of the biosynthesized Ag-NPs were demonstrated using XRD patterns. The XRD diffractogram depicted in Figure 4B shows four distinguished Bragg’s reflection planes at (111), (200), (220), and (311), which correspond to the 2θ values of 38.24, 44.26, 64.36 and 77.34, respectively. Analysis of the XRD profile was compared with the joint committee on powder diffraction standards (JCPDS) (No. 04-0783) [40]. The XRD study proved that the particles in the sample were silver nanoparticles, and the lattice planes were characteristics of the face-centered cubic (FCC) silver crystals. The mean crystalline size (D) of the Ag-NPs was determined from the diffractogram using the Debye–Scherrer’s formula, the mean crystalline size of the biogenic silver nanoparticles was ~28 nm. The absence of other peaks in the XRD spectra indicated the purity of the biosynthesized Ag-NPs. Similarly, Al-Bahrani et al. [44] reported the biogenic synthesis of polydisperse silver nanoparticles with an average size of 28 nm.

### 2.4. Antibacterial Activity of Ag-NPs Produced by the Endophytic S. laurentii R-1

The biocidal properties of the biocolloidal Ag-NPs suspension were evaluated based on zones of inhibition (mm). Ag-NPs were proven to be a promising and effective antibacterial agent in a dose-based mode against selected pathogens involving Gram-positive and Gram-negative bacteria. The antibacterial activity of nanoscaled silver particles was previously inferred by the formation of growth inhibition zones around an agar well [45]. The actinobacterial biomass filtrate (as a control) was checked for their activities as antimicrobial agents and did not exhibit any activities against the tested pathogenic bacteria, indicating their absence of active antibacterial metabolites.

**Figure 5:**
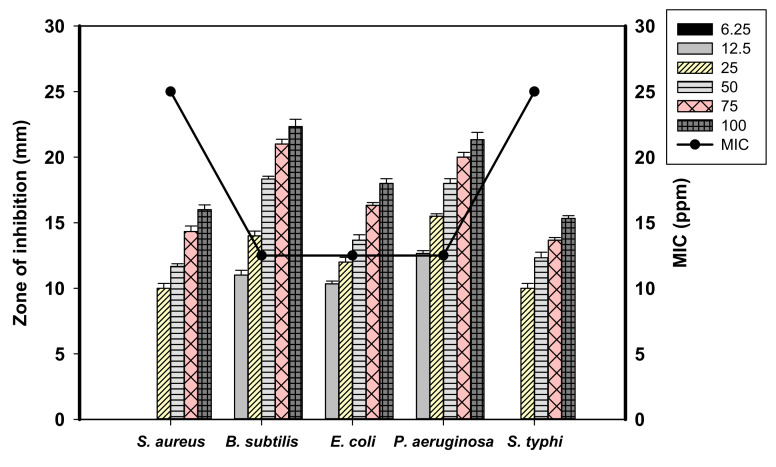
The antibacterial activity of Ag-NPs synthesized by *S. laurentii* R-1 against *Staphylococcus aureus*, *Bacillus subtilis*, *Pseudomonas aeruginosa*, *Escherichia coli* and *Salmonella typhimurium*.

The bio-silver nanoparticles (12.5 ppm) were efficient against the Gram-positive pathogen *Bacillus subtilis* ATCC 6633 and the Gram-negative pathogens *Pseudomonas aeruginosa* ATCC 9022, *Escherichia coli* ATCC 8739 with zones of inhibition (ZOIs) of 11 ± 0.89, 12.66 ± 0.52, and 10.33 ± 0.51 mm, respectively (Figure 5). Doubling the Ag-NPs concentration (25 ppm) conferred better efficiency, and the ZOIs increased to 14 ± 0.9, 15.5 ± 0.5, and 12 ± 0.9 mm for the pathogens *B. subtilis*, *P. aeruginosa*, and *E. coli*, respectively. Moreover, the Gram-positive pathogen *Staphylococcus aureus* ATCC 6538 and the Gram-negative pathogen *Salmonella typhimurium* ATCC 14028 manifested a positive response for silver nanoparticles at 25 ppm, with recorded ZOIs of 10 ± 0.9 and 10 ± 0.47 mm, respectively. The biocolloidal silver (50 ppm) demonstrated a pronounced activity against all pathogens, recording ZOIs of 11.6 ± 0.5, 18.3 ± 0.5, 13.6 ± 1, 18 ± 0.9, and 12.3 ± 1 mm corresponding to *S. aureus*, *B. subtilis*, *P. aeruginosa*, *E. coli*, and *S. typhimurium* respectively.

The Ag-NPs suspension (75 ppm) exhibited enhanced activity, and recorded the ZOIs of 14.3 ± 1.0, 21 ± 0.9, 16.3 ± 0.5, 20 ± 0.9, and 13.6 ± 0.5 mm corresponded to the pathogenic bacteria *S. aureus*, *B. subtilis*, *P. aeruginosa*, *E. coli*, *and S. typhimurium*, respectively. The highest level of inhibition listed for the maximum bio silver concentration (100 ppm) with ZOIs of 16 ± 0.9, 22.3 ± 1.3, 18 ± 0.9, 21.3 ± 1.2, and 15.3 ± 0.5 mm corresponded to the pathogens *S. aureus*, *B. subtilis*, *P. aeruginosa*, *E. coli*, and *S. typhimurium*, respectively (Figure 5). Silver nanoparticles derived from the *Streptomyces xinghaiensis* OF1 strain with inhibitory activity against pathogenic fungi and bacteria, including *S. aureus, B. subtilis*, *P. aeruginosa*, and *E. coli* was reported [46].

Bio-silver nanoparticles derived from actinobacteria previously demonstrated considerable potential as antibacterials [47]. The antibacterial efficacy of Ag-NPs could be attributed to the binding of silver with the bacterial cell wall through electrostatic attraction between the positive charge of Ag-NPs and the negative charge of the cell wall. This electrostatic attractions destroys the selective permeability via the reacting of Ag-NPs with sulfur and phosphorylated proteins located in the bacterial cell wall causing partial dissolution of the cell membrane [48]. Nanoparticles enter the cell and causes the liberation of Ag^+^, which causes enhancement of the reactive oxygen species (ROS) that destroys the enzymes involved in cellular respiration and are ultimately responsible for cell death [49]. In the same context, Al-Dhabi et al. [50] reported the extracellular synthesis of Ag-NPs from *Streptomyces parvus* with promising antibacterial activity against diverse pathogens involving *S. aureus*, *S. epidermidi*, *E. faecalis*, *B. subtilis*, *P. aeruginosa*, and *E. coli*.

The results of our study showed the efficacy of bio silver nanoparticles at low concentrations of 25 ppm; thus, these NPs could be used as a broad-spectrum nano bactericidal agent. The minimum inhibitory concentration (MIC) values and the corresponding inhibition zones of the biosynthesized Ag-NPs against pathogenic bacteria are specified and listed in Table 1.

### 2.5. In-Vitro Cytotoxicity of Ag-NPs against Normal and Cancer Cells

Recently, Ag-NPs have attracted great attention in developing new techniques for diagnosing and treating cancer [51]. Interestingly, biologically manufactured nanoparticles are more active than those synthesized by chemical or physical methods [36]. Accordingly, this study was designed to investigate the toxicity of biogenic nanoparticles against human cancer cells. For this reason, an 3-(4,5-dimethylthiazole-2-yl)-2,5-diphenyl tetrazolium bromide (MTT) assay was conducted to evaluate the cytotoxic activities of Ag-NPs obtained from *Streptomyces laurentii* against the monkey’s Vero normal cells and Caco-2 cells for human colon cancer.

MTT is an accurate colorimetric screening method commonly used to estimate cellular toxicity as well as cell viability and proliferation. The microscopic examination showed that the silver nanoparticles were toxic to eukaryotic cells due to their efficacy regarding the loss of the mono layer, which characterized the epithelial cells. The response of cloned cells to nanoparticles was concentration-dependent. The time, size, and dose-dependent cytotoxic effects of silver nanoparticles (especially particles of size ≤10 nm) were recently reported [52].

**Figure 6:**
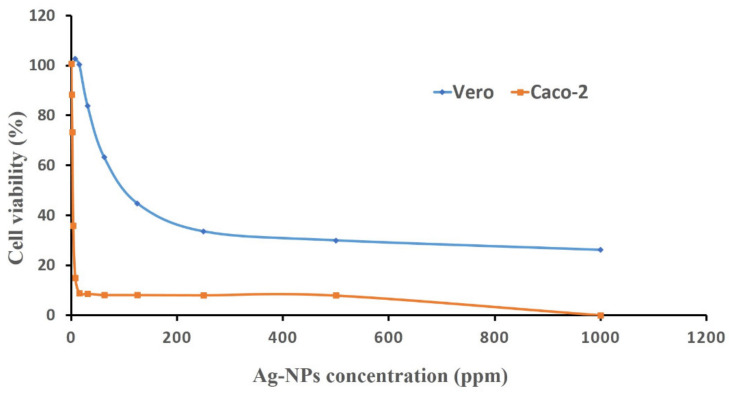
The cytotoxic activity of Ag-NPs derived from *Streptomyces laurentii* R-1 against Vero and Caco-2 cell lines.

Our investigations revealed that cloned cells treated with nanoparticles lost their ability to adhere, as demonstrated by a partial or complete collapse of the mono layer integrity along with floating, shrinking, and the cells becoming spherical and granular. Comparable morphological changes were recorded by treating the normal Wi 38 and the cancerous Caco-2 cell lines with biologically created selenium nanoparticles, where the cells lost their shape and monolayer integrity in addition to shrinking, rounding, or cell granulation [53]. The values of bio fabricated Ag-NPs required for 50% cell mortality (IC_50_) were calculated from the curve (Figure 6). The IC_50_ for normal Vero cells (158 ± 9.53 ppm) was much greater than the corresponding value for cancer Caco-2 cells (4.66 ± 0.21 ppm).

Consequently, the biogenic Ag-NPs demonstrated selective toxicity towards cancer cells (Caco-2), as they demonstrated an anti-proliferative effect on cancer cells at low concentrations. On the contrary, nanoparticles affected the survival and population of normal cells (Vero) only if used in very high concentrations, this window can be used medically for the selective activity of nanoparticles against cancer cells. Consistent with these results, Hamouda et al. [43] reported the cytotoxic potential of bio synthesized Ag-NPs against the human breast cell line (MCF-7) and human colon (HCT-116) cancer cell line, recording IC_50_ values of 6.147 and 5.369 µg/mL, respectively. Silver nanoparticles may cause cell death by the interaction of the liberated silver ions with the cellular components, resulting in the production of ROS, and oxidative stress that ends in apoptic death [54].

This interaction decreases adenosine tri-phosphate (ATP) synthesis and damages cellular protein functioning; enzymes, such as protein kinases, prohibit cell repair [55]. Another cytotoxic mechanism of Ag-NPs is the Trojan-horse; these include exposing cells to elevated levels of silver ions after the entry of Ag-NPs into cells. Silver ions also bind directly to RNA polymerase and block its activity [56]. From an industrial overview, NPs are effective in treating cotton fabrics with a concentration that affects the cancerous cell and stays safe to a normal cell, in addition to their activity against human pathogenic bacteria. Therefore, the current study suggests that the biosynthesized Ag-NPs concentration of 100 ppm is appropriate for cotton fabric treatment as a safe dose.

### 2.6. Application of Biologically Manufactured Silver Nanoparticles

#### 2.6.1. Loading Silver Nanoparticles on Cotton Fabrics

The pieces of cloth were washed, loaded with bio colloidal Ag-NPs (100 ppm) using a pad-dry curing method. This concentration is safe for normal mammalian cells, according to our cytotoxicity examination. Immersing and washing fabrics with water before the treatment forms a negative surface charge on the textile fibers, which can then electrostatically attract the positively charged silver nanoparticles [57]. The surface topography of the samples was analyzed using scanning electron microscopy (SEM). Microscopic micrographs of the pristine cloth showed a pure interlocking fibrous structure with a smooth surface, free from contaminating particles.

While nano-treated fabrics appeared to retain their structure, their surface was coated with Ag-NPs that merged with the textile fibers and dispersed uniformly across the surface of the tissue. Recently, Haiting et al. [58] authenticated the deposition of Ag-NPs evenly distributed across the surface of a treated cotton cloth. In our study, the small size of the Ag-NPs (7–28 nm) gave rise to their successful adhesion to the tissue surface, since the smaller the particles, the more deeply they penetrated the tissues and tightly adhered to the cotton fibers.

**Figure 7:**
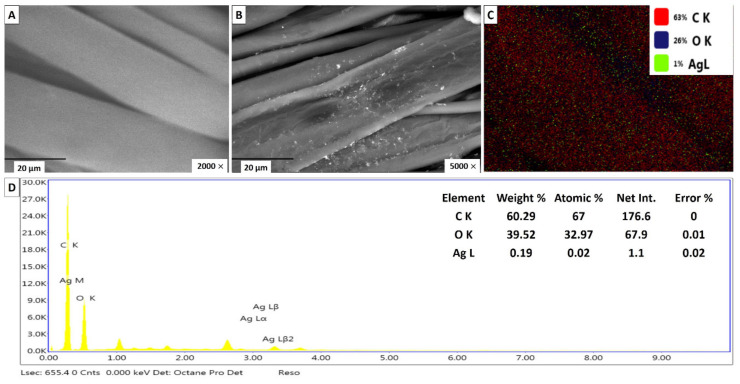
Scanning electron microscope (SEM) image (**A**) Blank cotton fabric; (**B**) treated cotton fabrics with Ag-NPs 100 ppm; (**C**) mapping picture of the surface of treated fabrics with Ag-NPs; and (**D**) energy dispersive X-ray spectroscopy (EDX) of a treated sample with elemental analysis of the Ag-NPs contents.

The energy dispersive X-ray spectroscopy (EDX) spectra provided an elemental chemical profile for the textile surface; in addition to the peaks of O and C, Ag-based fabric samples displayed an incoming peak at around ca. 3 keV, which could be assigned to the nanoscaled silver. The nanosized colloidal silver represented 1% of the total tissue surface elements, or approximately 0.19% of the total weight of these elements. These results proved the deposition of fine silver particles on the fabric surface (Figure 7). Consistent with the current study, Othman et al. [59] reported the successful use of biogenic colloidal silver as a nano-finishing agent for antimicrobial textiles. Their EDX analysis confirmed the presence of elemental silver with the strong peaks recorded at 3.0 keV. The results obtained showed that the Ag-NPs dispersion was successfully incorporated into the surface of the treated fabric with high-density coverage, indicating the possibility of using silver particles as a nano-finishing agent for multifunctional textiles.

#### 2.6.2. Antibacterial Activity of Nano-Treated Fabrics

Generally, textiles that come into contact with human skin provide a warm and moist environment for microorganisms. Sweaty fabrics are an ideal environment for bacterial growth and proliferation [60,61]. Hence, functionalizing fabrics with Ag-NPs for antimicrobial properties is an interesting aspect of processing garments and clinical dressings. Additionally, Ag-NPs provide the finished fabrics with water and oil repellent properties [62]. Thus, this prevents the Ag-fabric from becoming wet and gathering moisture, thereby preventing microbial growth.

The antibacterial potential of nano-finished fabrics against selected bacterial pathogens was evaluated using inhibition zones. Our results demonstrated that the raw cotton samples did not have any antibacterial activity either before or after washing. In contrast, although nanoparticles were present in a small concentration, their uniform distribution across the surface of the finished fabrics imparted a broad-spectrum activity. Ag-fabrics displayed the maximum inhibition zone against *B. subtilis* (2.63 ± 0.15 mm), while a lesser inhibition zone was recorded against *P. aeruginosa* (1.23 ± 0.05 mm). The functionalized fabrics exhibited a considerable activity against *S. aureus* and *E. coli*, registering the inhibition zones of 1.62 ± 0.15 and 1.91 ± 0.07 mm, respectively.

Evidently, smaller nanoparticles have a large surface area, which increases their degree of contact with the bacteria, leading to the formation of larger inhibition zones [63]. Washing durability is an indispensable and essential factor for the application of antibacterial Ag-fabrics. Although the antibacterial activity of treated tissue decreases by washing, the current study demonstrated that even after 10 cycles of repeated washing, the fabric retained the nanoscaled silver cover, and was still able to inhibit bacterial growth (Table 2). However, Ag-NPs could separate from the fabric due to high temperatures and mechanical vibration during washing [52]. Therefore, the treated fabric lost its activity through successive washing. Consistent with our results, Othman et al. [59] revealed the application of fungal induced Ag-NPs for finishing durable antimicrobial fabrics and reported the activity of Ag-fabrics against *Escherichia coli, Bacillus mycoides* and *Candida albicans*. They added that the cloth retained the silver nanomaterials even after five repeated wash cycles.

## 3. Materials and Methods

### 3.1. Plant Sample Collection and Isolation of Endophytic Actinomycetes

Root samples of a flourishing medicinal plant *Achillea fragrantissima* (Forssk.) Sch. Bip. (Family *Asteraceae*) were carefully collected from area of Selebat (28.545493 N, and 33.933707 E), Saint Katherine, South Sinai, Egypt. The collected samples were stored in sterile bags, transported to the laboratory in the icebox, and subjected to selective isolation methods within 24 h of collection. The botanical identification of plant sample was achieved in the herbarium of Botany and Microbiology Department of Al-Azhar University. Surface sterilization of the root samples was performed according to Fouda et al. [64] and ALKahtani et al. [14]. The sterilized roots were cut into small pieces and deposited on starch casein agar (SCA) [65] containing nystatin 25 µg mL^−^^1^ and nalidixic acid 50 µg mL^−^^1^. The inoculated plates were incubated for 30 days at 28 °C and observed daily for actinobacterial growth. Pure cultures were maintained in SCA slants for further study.

### 3.2. Genotypic Identification of Endophytic Actinobacteria

A single colony of the selected bacterial species was picked and washed in saline solution (NaCl 0.085%). The genomic DNA was extracted using a GeneJET Genomic DNA Purification Kit (Thermo K0721) according to the manufacturer’s criteria and was used as a template for PCR amplification. The 16S-rRNA gene was amplified using Maxima Hot Start PCR Master Mix (Thermo K1051) in 20 mL reactions using the universal primers of 27 f (50-AGAGTTTGATCCTGGCTCAG-30) and p1492r (50-TACGGCTACCTTGTTACGACT) [66]. The amplification products were checked by electrophoresis on 1% agarose gels and were commercially sequenced by the GATC Company using ABI 3730xl DNA sequences. The bacterial 16S sequences in this study were deposited in GenBank under the accession number of MT534273. The obtained sequences and those of their most closely related taxa retrieved from GenBank were aligned using the CLUSTAL X program [67]. The phylogenetic distances were calculated with Kimura’s two-parameter model [68], and evolutionary trees were inferred using neighbor-joining [69]. A phylogenetic tree was developed using MEGA v6.1 software, with the confidence tested by a bootstrap analysis (1000 repeats).

### 3.3. Biosynthesis of Silver Nanoparticles (Ag-NPs) Using Endophytic Actinomycetes

#### 3.3.1. Biomass Preparation

Three agar plugs (6 mm diameter) of a freshly grown culture of the isolated endophytic actinomycetes were inoculated into autoclaved conical flasks containing 100 mL of starch nitrate broth (SNB) medium. The flasks were incubated for 5 days at 30 °C ± 2 in a rotating orbital shaker (150 rpm). At the end of the incubation period, the actinomycetes biomass was harvested by passing through four layers of wool fabric.

#### 3.3.2. Preparation of Biomass Filtrate

The obtained actinomycetes biomass was washed thrice with sterile distilled water to remove any adhering medium components, followed by suspension in 100 mL distilled water and maintained at 30 ± 2 °C for 72 h. Then, the mixture was filtered through Whatman filter paper No.1. Ultimately, the filtrate was collected to be ready for the bioreduction of AgNO_3_ (precursors for Ag-NPs).

#### 3.3.3. Nano Silver Synthesis

We added 1 mM aqueous solution of AgNO_3_ to 100 mL of the collected biomass filtrate and incubated overnight at 35 °C in static and dark conditions [15]. The pH of the mixture was adjusted to 9 by 1 M NaOH, which we added drop-wisely under stirring conditions. The resultant yellowish brown Ag-NPs was subjected to oven-drying at 100 °C for 24 h. The actinomycetes biomass filtrate, as well as AgNO_3_ were running with the experiment as negative controls.

### 3.4. Characterization of Biosynthesized Silver Nanoparticles

#### 3.4.1. UV-Vis Spectrophotometry

UV-Vis spectrophotometry (JENWAY 6305 Spectrophotometer) was used for assessing the absorption properties of the biogenic synthesized Ag-NPs. The mixtures containing Ag-NPs were examined in the wavelength range of 300–700 nm (10 nm intervals) using quartz cuvettes (1 cm path length). The presence of Ag-NPs can be observed by allocating a peak in the range of 400 to 500 nm. The endophytic actinomycetes biomass filtrate was used as a blank for the JENWAY spectrophotometer to confirmed the color change.

#### 3.4.2. Transmission Electron Microscopy

The shape and size of biogenic Ag-NPs fabricated by the isolated actinobacterial endophyte isolate was verified by transmission electron microscopy (TEM- JEOL 1010 Japan). For TEM measurements, the drop of biogenic Ag-NPs suspension was scooped on a copper TEM grid with coated carbon [23].

#### 3.4.3. FT-IR Analysis

Functional groups of the actinomycetes biomass filtrate and their role in the reduction, capping, and stabilization of biosynthesized Ag-NPs were characterized by Fourier transform infrared (FT-IR) spectroscopy (Agilent system Cary 630 FTIR model) at the range of 400–4000 cm^−1^.

#### 3.4.4. X-ray Diffraction Analysis

XRD analysis of Ag-NPs was performed using Shimadzu Scientific Instruments (SSI), Kyoto, Japan. X-ray Diffraction patterns of silver nanoparticles were obtained with the XRD-6000 series, including stress analysis, residual austenite quantitation, crystallite size/lattice strain, crystallinity calculations, materials analysis via overlaid X-ray diffraction patterns with the Shimadzu apparatus using a nickel-filter, and Cu-Ka targets. The estimated particles size by XRD analysis was performed with Scherrer’s formula as follows:

D = 0.94λ/β cos *θ* (β was estimated by Origin 2017 software)

where D is the mean particle size, 0.94 is the Scherrer’s constant, λ is the X-ray wavelength, β is the half of the maximum intensity, and *θ* is the Bragg’s angle.

### 3.5. Antibacterial Activity of Ag-NPs Produced by the Endophytic Actinobacterial Isolate

The antibacterial activity of the biologically manufactured Ag-NPs was evaluated against five pathogens, including; Gram-positive strains (*Staphylococcus aureus* ATCC 6538 and *Bacillus subtilis* ATCC 6633) and Gram-negative strains (*Pseudomonas aeruginosa* ATCC 9022, *Escherichia coli* ATCC 8739, and *Salmonella typhimurium* ATCC 14028). The selected bacterial strains were cultured on nutrient agar media for 24 h at 37 °C. Bacterial suspensions of 1.5 × 10^8^ CFU/mL were separately prepared in sterile saline solution (0.85% v/v), seeded into Muller Hinton agar media, and poured aseptically into sterilized petri plates. Three wells (0.8 cm diameter) were cut in the solidified seeded media, and each well was filled with 100 µL of Ag-NPs (Ag-NPs suspended in distilled water). After loading with Ag-NPs, Muller Hinton plates are set in the refrigerator for 1 h. and after that, incubated for 24 h at 37 °C. The potentially clear zones around the wells were measured (mm) and recorded. Ag-NPs were prepared in different concentrations (6.25, 12.5, 25.0, 50.0, and 75.0 ppm), and assessed individually to determine the minimum inhibitory concentration (MIC) against each tested organism [70].

### 3.6. In Vitro Cytotoxicity of Ag-NPs against Normal and Cancer Cells

#### 3.6.1. Cell Lines Culture used

The normal Vero cells (kidneys of African green monkey) and human colorectal adenocarcinoma cells (Caco-2) were obtained from American type culture collection (ATCC).

#### 3.6.2. Investigate the Cell Morphology

Cells were grown in 96-well microtiter plates at 1 × 10^5^ cells/well, treated with double fold dilution (1000–0.48 ppm) of the biosynthesized Ag-NPs. The treated cells were incubated for 48 h and observed by an inverted microscope (Nikon, Japan) for cytotoxic analysis.

#### 3.6.3. MTT Assay

The potential cytotoxicity of the biogenic Ag-NPs was evaluated using an MTT [3-(4,5-dimethylthiazol-2-yl)-2,5-diphenyl tetrazolium bromide] assay using normal Vero cells and human colorectal adenocarcinoma cells (Caco-2). Briefly, the cells were cultured in 96-well microtiter plates at 1 × 10^5^ cells/well and treated with double fold dilution (1000–0.48 ppm) of the biosynthesized Ag-NPs and incubated for 48 h. at 37 °C. MTT (5 mg mL^−1^ in phosphate-buffered saline) was then added to each well and incubated at 37 °C for 1–5 h, 5% CO_2_. Then purple formazan crystals were formed, which were further dissolved by adding DMSO (10%). The plates were agitated using a plate shaker for 30 min in dark conditions. Ultimately, the color intensity of samples was measured at 560 nm using a multi well ELISA plate reader [71]. The cell viability percentage was calculated as follows:

Cell viability % = (sample absorbance/control absorbance) × 100.

### 3.7. Application of Biologically Manufactured Silver Nanoparticles

#### 3.7.1. Loading Silver Nanoparticles on Cotton Fabrics

The cotton cloth was cut into pieces (15 × 30 cm); these pieces were washed with warm water and left to dry. The cotton fabric pieces were padded with Ag-NPs solution at a specific safe concentration (based on in-vitro cytotoxic results). For the successful treatment of textiles with colloidal silver, the solution was constantly stirred. All samples were immersed in this colloidal bath for one minute and then squeezed up to 100% wet pick up with a laboratory pad at a steady pressure. Samples were dried at 70 °C for 3 min, and then ironed at 150 °C for 2 min. The following treatments were performed: (1) Non-treated fabrics as a control, (2) Ag-NP-treated fabrics, and (3) Ag-NP-treated fabrics after repeated to 5 and 10 washing cycles. Washing was performed using a set of warm water machines (40–60 °C) containing 2% sodium carbonate. After each washing cycle (45 min), the fabrics were dried in a dryer at 80 °C.

#### 3.7.2. Surface Properties of the Nanocoated Fabrics

Energy dispersive X-ray spectroscopy (EDX) in combination with SEM (Jeol–JSM-5400, Japan) was used to determine the morphological and surface characterization as well as the elemental composition of the treated fabrics.

#### 3.7.3. Antibacterial Activity of Nano-Treated Fabrics

The antibacterial efficacy of the nano-treated fabrics was evaluated against selected pathogenic Gram positive (*Staphylococcus aureus* ATCC 6538 and *Bacillus subtilis* ATCC 6633) and Gram-negative strains (*Pseudomonas aeruginosa* ATCC 9022 and *Escherichia coli* ATCC 8739). Squares of the fabric samples were aseptically prepared and placed on the solidified Muller–Hinton agar plates that were pre-seeded with each bacterial pathogen. After incubation for 24 h, the plates were observed and the diameters (mm) of the inhibition zones around each sample were recorded. The experiment was conducted with these treatments; (1) Non-treated fabrics as a control, (2) Ag-NP-treated fabrics, and (3) Ag-NP-treated fabrics that were washed for 5 or 10 repeated washing cycles.

## 4. Statistical Analysis

All results presented in this study are the means of three independent replicates. The data were subjected to analysis of variance (ANOVA) by statistical package SPSS v17. The mean difference comparison between the treatments was analyzed by the Tukey HSD (Honestly Significant Difference) test at a significance level of *p* ≤ 0.05.

## 5. Conclusions

This study presented the successful bio-fabrication of silver nanoparticles in an economical and environmentally safe manner. The medicinal plant of *Achillea fragrantissima* was explored to isolate the endophytic *Streptomyces laurentii* which mediated the biogenic synthesis of the stable and spherical Ag-NPs of the 7–28 nm size range. These particles showed wide spectrum activity against the representative clinical pathogens (*S. aureus, B. subtilis, P. aeruginosa, E. coli,* and *S. typhimurium*), as well as anti-tumor activity against the human cancer colon cell line. We applied biocolloidal silver as nano-finishing materials for multifunctional textile fabrics. EDX and SEM analysis proved the homogenous loading of Ag-NPs onto the fabric surface was achieved. The Ag-based fabrics exhibited durable broad-spectrum antibacterial activity that lasted after 10 cycles of repeated washing. These treated fabrics are recommended to be used clinically as textiles or dressings.

## Figures and Tables

**Table 1:** The inhibition zones and minimum inhibitory concentration (MIC) values of the biosynthesized Ag-NPs against pathogenic bacteria.

Bacterial Pathogens	Inhibition Zone Diameter (mm)	MIC (ppm)
*S. aureus* ATCC 6538	10 ± 0.9	25
*B. subtilis* ATCC 6633	11 ± 0.89	12.5
*P. aeruginosa* ATCC 9022	12.66 ± 0.52	12.5
*E. coli* ATCC 8739	10.33 ± 0.51	12.5
*S. typhimurium* ATCC 14028	10 ± 0.47	25

**Table 2:** The effects of repeated washing on the antibacterial properties of silver nanoparticles finished fabrics with the qualitative assessment method.

Number of Washing Cycles	Clear Zone (mm)			
	*S. aureus*	*B. subtilis*	*P. aeruginosa*	*E. coli*
Before washing	1.62 ± 0.15 ^a^	2.63 ± 0.15 ^a^	1.23 ± 0.05 ^a^	1.91 ± 0.07 ^a^
After 5 cycles	0.97 ± 0.12 ^b^	2.06 ± 0.11 ^b^	0.84 ± 0.05 ^b^	1.71 ± 0.05 ^b^
After 10 cycles	0.69 ± 0.1 ^c^	1.47 ± 0.3 ^c^	0.57 ± 0.1 ^c^	0.91 ± 0.07 ^c^

Different letters between columns denote that mean values are significantly different (*p* ≤ 0.05) LSD test.
